# High-sensitivity troponin I with or without ultra-sensitive copeptin for the instant rule-out of acute myocardial infarction

**DOI:** 10.3389/fcvm.2022.895421

**Published:** 2022-08-09

**Authors:** Fabrizio Ricci, Johannes T. Neumann, Nicole Rübsamen, Nils A. Sörensen, Francisco Ojeda, Ivana Cataldo, Tanja Zeller, Sarina Schäfer, Tau S. Hartikainen, Maria Golato, Stefano Palermi, Marco Zimarino, Stefan Blankenberg, Dirk Westermann, Raffaele De Caterina

**Affiliations:** ^1^Department of Neuroscience, Imaging and Clinical Sciences, “G. d’Annunzio” University, Chieti-Pescara, Italy; ^2^Department of Clinical Sciences, Lund University, Malmö, Sweden; ^3^Casa di Cura Villa Serena, Città Sant’Angelo, Pescara, Italy; ^4^Department of General and Interventional Cardiology, University Heart Center Hamburg, Hamburg, Germany; ^5^German Center for Cardiovascular Research (DZHK), Partner Site Hamburg/Kiel/Lübeck, Hamburg, Germany; ^6^Unit of Clinical Pathology, SS. Annunziata University Hospital, Chieti, Italy; ^7^Public Health Department, University of Naples Federico II, Naples, Italy; ^8^Cardiology Division, Pisa University Hospital and University of Pisa, Pisa, Italy

**Keywords:** high-sensitivity cardiac troponin, copeptin, coronary artery disease, emergency department, myocardial infarction

## Abstract

**Background:**

The instant, single-sampling rule-out of acute myocardial infarction (AMI) is still an unmet clinical need. We aimed at testing and comparing diagnostic performance and prognostic value of two different single-sampling biomarker strategies for the instant rule-out of AMI.

**Methods:**

From the Biomarkers in Acute Cardiac Care (BACC) cohort, we recruited consecutive patients with acute chest pain and suspected AMI presenting to the Emergency Department of the University Medical Center Hamburg-Eppendorf, Hamburg, Germany. We compared safety, effectiveness and 12-month incidence of the composite endpoint of all-cause death and myocardial infarction between (i) a single-sampling, dual-marker pathway combining high-sensitivity cardiac troponin I (hs-cTnI) and ultra-sensitive copeptin (us-Cop) at presentation (hs-cTnI ≤ 27 ng/L, us-Cop < 10 pmol/L and low-risk ECG) and (ii) a single-sampling pathway based on one-off hs-cTnI determination at presentation (hs-cTnI < 5 ng/L and low-risk ECG). As a comparator, we used the European Society of Cardiology (ESC) 0/1-h dual-sampling algorithm.

**Results:**

We enrolled 1,136 patients (male gender 65%) with median age of 64 years (interquartile range, 51–75). Overall, 228 (20%) patients received a final diagnosis of AMI. The two single-sampling instant rule-out pathways yielded similar negative predictive value (NPV): 97.4% (95%CI: 95.4–98.7) and 98.7% (95%CI: 96.9–99.6) for dual-marker and single hs-cTnI algorithms, respectively (*P* = 0.11). Both strategies were comparably safe as the ESC 0/1-h dual-sampling algorithm and this was consistent across subgroups of early-comers, low-intermediate risk (GRACE-score < 140) and renal dysfunction. Despite a numerically higher rate of false-negative results, the dual-marker strategy ruled-out a slightly but significantly higher percentage of patients compared with single hs-cTnI determination (37.4% versus 32.9%; *P* < 0.001). There were no significant between-group differences in 12-month composite outcome.

**Conclusions:**

Instant rule-out pathways based on one-off determination of hs-cTnI alone or in combination with us-Cop are comparably safe as the ESC 0/1 h algorithm for the instant rule-out of AMI, yielding similar prognostic information. Instant rule-out strategies are safe alternatives to the ESC 0/1 h algorithm and allow the rapid and effective triage of suspected AMI in patients with low-risk ECG. However, adding copeptin to hs-cTn does not improve the safety of instant rule-out compared with the single rule-out hs-cTn at very low cut-off concentrations.

## Introduction

A fast and accurate diagnosis of acute myocardial infarction (AMI) is key to ensure a swift treatment initiation, and is associated with improved clinical outcome (1–3). Because acute chest pain is an extremely common presentation in Emergency Departments (ED), entailing millions of annual ED visits worldwide and with 10–15% of them eventually having a final diagnosis of AMI (4, 5), rapid, safe and effective rule-out strategies are highly desirable to improve resource allocation and speed-up the diagnostic work-up. This is particularly valuable in the setting of non-ST elevation AMI, where ECG findings are often non-diagnostic. The European Society of Cardiology (ESC) recommends the use of high-sensitivity cardiac troponin (hs-cTn) on admission and after 1 h if a hs-cTn test with a validated 0/1 h algorithm is available (Class I, Level B), and has further endorsed the use of a 0/2 h algorithm (Class I, Level B). In both cases cut-off values are assay-specific and below the 99th percentile of normal values (3). Strategies using a single admission rule-out have also been suggested, yet are still investigational (6, 7). Two single admission rule-out strategies have currently been proposed: one based on a single sampling of hs-cTn and using low-cut-off concentrations (2, 8, 9), the other based on the single simultaneous measurement of two analytes, cTn and copeptin. Evidence from the large randomized BIC-8 trial (10), observational studies (11–14), real-world data (15), and two meta-analyses (16, 17) have indeed established the usefulness and safety of copeptin – the C-terminal fragment of vasopressin precursor hormone, currently interpreted as a quantitative marker of endogenous stress – in combination with cTn for the instant rule-out of AMI (3, 18).

The evaluation of diagnostic tests should be no different than other interventions. The ultimate proofs of safety and, in particular, efficiency rely on randomized controlled trials (RCT) also in this area (19), and this process has been now applied also to hs-cTn and copeptin (10, 20–23). Nevertheless, among available protocols tested within an RCT, which strategy is most valuable for the instant rule-out of AMI is still unknown.

In this study, we compared safety, effectiveness and prognostic performance of two currently competing single-sampling diagnostic strategies with reference to the ESC 0/1-h algorithm: (i) a single-sampling dual-marker strategy combining 2nd-generation ultrasensitive copeptin (us-Cop), hs-cTnI (99th percentile threshold) and low-risk ECG; (ii) a single-sampling hs-cTnI strategy combining one-off hs-cTnI determination at presentation with a threshold of less than < 5 ng/L (8, 9) and low-risk ECG.

## Materials and methods

### Study population

**FIGURE 1:**
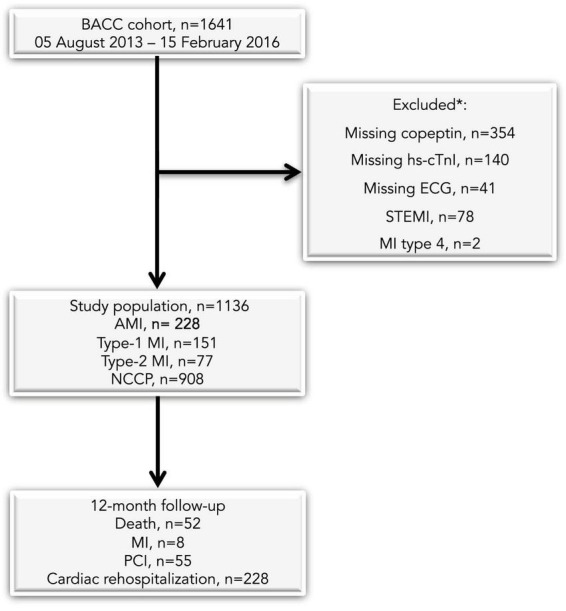
Selection process of the study population. AMI, acute myocardial infarction; NCCP, non-coronary chest pain; MI, myocardial infarction; STEMI, ST-elevation myocardial infarction; PCI, percutaneous coronary intervention. *More than one exclusion criterion can apply to a single patient.

For the present analysis, we used data from the Biomarkers in Acute Cardiac Care (BACC) study cohort. The study had been approved by the local Ethics Committee (Ärztekammer Hamburg, Germany) and registered at www.clinicaltrials.gov (NCT02355457), and its design complied with the Declaration of Helsinki. All patients provided written informed consent. The study population has been previously described (7). We prospectively recruited patients with suspected AMI presenting at the ED or the Chest Pain Unit at the Hamburg-Eppendorf University Hospital between July 19th, 2013 and April 10th, 2016. Exclusion criteria were: (i) age < 18 years; (ii) ST-segment elevation MI; (iii) AMI type 4; (iv) missing copeptin and hs-cTnI values; (v) missing ECG information; (vi) cardiac arrest survivors. The final study population consisted of 1,136 individuals (Figure 1).

### Study design

All patients underwent the local clinical routine according to current ESC Guidelines, including an immediate ECG, ECG monitoring, serial measurement of high-sensitivity troponin T (hs-TnT, Elecsys^®^, Roche Diagnostics) at admission and after 3 h, as well as further imaging techniques (e.g., echocardiography or angiography) when clinically demanded (1). Decision on the specific treatment (discharge, further observation, or admission) was left to the treating physicians. The ECG was interpreted by the ED physician and reassessed by a cardiologist for adjudication of the final diagnosis. The ECG was considered “low-risk” when no ST-depression (≥ 0.05 mV), T-wave inversion (≥ 0.1 mV), ST-elevation (≥ 0.1 mV or ≥ 0.2 mV in V2/3), arrhythmias, atrioventricular block or left bundle branch block were observed, as previously reported (7). Previous ECGs were not available for the adjudication. The assessment of other clinical parameters and cardiovascular risk factors has been reported before (24).

### Adjudicated final diagnosis

Adjudication of the final diagnosis was done by two physicians separately and in a blinded fashion. In cases of disagreement, a 3rd physician was called-upon. The final gold-standard diagnosis was based on all available clinical, ECG, imaging and laboratory parameters (excluding hs-cTnI and us-Cop). Acute MI was defined in accordance with the Third Universal Definition of MI (25), but also consistent with the current Fourth Universal Definition (26). Briefly, MI was diagnosed when there was evidence of myocardial necrosis in combination with clinical symptoms suggestive of myocardial ischemia. According to current recommendations, acute myocardial injury detected by a rising and/or falling pattern of cTn values above the sex-specific 99th percentile, was designated as MI when clinically thought to be due to myocardial ischemia. Absolute hsTnT changes were used to determine significant changes. A significant absolute change was defined as a rise or fall of hsTnT > 6 ng/L within 3 h. All other clinical occurrences were categorized as non-MI.

### Laboratory analyses

High-sensitivity cardiac troponin I was measured using the Architect immunoassay (ARCHITECT i1000SR, Abbott Diagnostics) at admission, and after 1 and 3 h. This specific assay has a limit of detection of 1.9 ng/L and a < 10% coefficient of variation at a concentration of 5.2 ng/L (27). In the general population the 99th percentile has been reported at 27 ng/L (28). Measurements were performed in part from fresh samples and in part in batches from samples that had been frozen at −80°C and never previously thawed. Copeptin measurements were performed retrospectively in batches of frozen admission samples using the BRAHMS copeptin proAVP automated immunofluorescent assay on the KRYPTOR Compact Plus system (Thermo Fisher Scientific, Hennigsdorf, Germany). This assay has a lower limit of detection of 0.9 pmol/L and a functional assay sensitivity of < 2 pmol/L. The direct measuring range was 0.9 to 500 pmol/L. For the analysis, we used a cut-off concentration of 10 pmol/L, as recommended by the ESC Study Group on Biomarkers in Cardiology of the Acute Cardiovascular Care Association (10).

### Follow-up

We followed-up all patients over 12 months to assess the cluster of all-cause death and MI as the primary composite outcome, and a secondary combined endpoint including all-cause death, MI, subsequent myocardial revascularization and cardiac rehospitalization. Patients were contacted by phone call, letter, through the general practitioner, or using medical records. In cases without any follow-up information, we contacted the local death register death. Overall 1,638 of 1,641 patients (99.8%) completed the 12-month follow-up (Figure 1).

### Diagnostic strategies

We investigated and compared two different early-time-point strategies to rule-out MI: (i) a single-draw, dual-marker algorithm based on the 99th percentile of hs-cTnI ≤ 27 ng/L, us-Cop < 10 pmol/L and a low-risk ECG; and (ii) a baseline hs-cTnI concentration < 5 ng/L in combination with a low-risk ECG, as a single-draw, single-marker diagnostic strategy (6, 8, 9). We compared the above two single-draw strategies with the ESC 0/1-h algorithm as the reference (1): here MI is ruled-out based on a baseline hs-cTnI concentration < 2 ng/L if chest pain onset > 3 h; or < 5 ng/L plus a delta of < 2 ng/L upon a serial sampling after 1 h.

### Statistical analysis

We described continuous variables as quartiles, and categorical variables as absolute numbers and percentages. For between-group comparisons we employed the Wilcoxon rank-sum test for continuous variables or the χ^2^ test for categorical variables. We compared safety [sensitivity and negative predictive value (NPV)] and effectiveness (percentage of patients referred to the rule-out) of the three algorithms with reference to the adjudicated final diagnosis of MI. To assess safety, we compared NPVs using the test described by Kosinski (29). We compared sensitivities by calculating 2-by-2 tables of patients with a diagnosis of MI only, and performing the exact McNemar tests on these tables as proposed by Trajman and Luiz (30). To assess effectiveness, exact McNemar tests on the 2-by-2 tables of all patients were used to test the agreement of the algorithms. Survival curves for those patients that were ruled-out by the different algorithms were produced using the Kaplan-Meier method. To compare survival curves, we used the grouped jackknife method (31) because patients could be included simultaneously into two curves (in case that they had been ruled-out by both algorithms). We considered a two-sided *P*-value of < 0.05 as statistically significant. We ran all statistical tests with the R statistical software, version 3.4.3.^1^

## Results

### Baseline demographics

Baseline characteristics of the study population are summarized in Table 1. We included 1,136 patients with a median age of 64 (interquartile range, 51–75) years. Overall, we adjudicated the final diagnosis of AMI in 228 (20%) patients. Patients with AMI were older, more often hypertensive and dyslipidemic, and presented with significantly higher values of us-Cop and worse renal function compared with the population without AMI.

**TABLE 1:** Baseline characteristics of the study cohort.

Characteristics	All (*N* = 1,136)	NCCP (*N* = 908)	AMI (*N* = 228)	*P*-value
Age (years)	64.0 (51.0, 75.0)	62.5 (49.0, 74.0)	69.0 (59.0, 77.0)	<0.001
Male (%)	738 (65.0)	584 (64.3)	154 (67.5)	0.40
BMI (kg/m^2^)	26.1 (23.6, 29.4)	26.0 (23.5, 29.3)	26.5 (23.6, 30.1)	0.24
Hypertension (%)	772 (68.3)	594 (65.7)	178 (78.4)	<0.001
Dyslipidemia (%)	453 (39.9)	335 (36.9)	118 (51.8)	<0.001
Diabetes (%)	152 (13.5)	112 (12.5)	40 (17.6)	0.056
Former smoker (%)	354 (31.2)	278 (30.7)	76 (33.3)	0.49
Current smoker (%)	276 (24.3)	216 (23.8)	60 (26.3)	0.49
History of CAD (%)	393 (34.6)	292 (32.2)	101 (44.3)	<0.001
Low-risk ECG (%)	675 (59.4)	586 (64.5)	89 (39.0)	<0.001
eGFR (ml/min/1.73 m^2^)	76.9 (59.6, 92.7)	80.0 (61.5, 94.0)	68.2 (52.0, 83.1)	<0.001
Symptom onset ≥ 6 h (%)	606 (57.5)	483 (57.8)	123 (56.2)	0.71
hs-cTnI 0 h (ng/L)	6.5 (3.0, 18.0)	4.9 (2.4, 10.3)	64.9 (16.1, 718.5)	<0.001
hs-cTnI 1 h (ng/L)	6.7 (2.9, 21.4)	5.1 (2.5, 10.1)	131.8 (31.8, 731.3)	<0.001
us-Cop 0 h (pmol/L)	6.8 (3.6, 17.9)	5.9 (3.3, 14.6)	11.5 (5.5, 35.7)	<0.001

AMI, acute myocardial infarction; BMI, body mass index; CAD, coronary artery disease; eGFR, estimated glomerular filtration rate; hs-cTnI, high-sensitivity cardiac troponin I; PCI, percutaneous coronary intervention; NCCP, non-coronary chest pain; us-Cop, ultrasensitive copeptin.

### Safety

**FIGURE 2:**
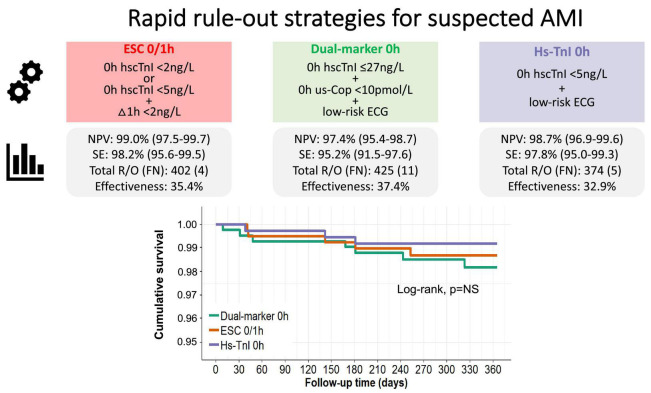
Diagnostic performance of instant rule-out strategies for suspected AMI and ESC 0/1-h algorithm with reference to serial 0/3-h sampling, and Kaplan-Meier analysis of the 12-month primary composite endpoint. AMI, acute myocardial infarction; FN, false negatives; NPV, negative predictive value; NS, not significant; R/O, rule-out; SE, sensitivity.

Diagnostic performance of the single-sampling pathways and of the ESC 0/1-h algorithm are reported in Table 2. The dual-marker strategy ruled out AMI with 95.2% (95%CI 91.5–97.6) sensitivity and 97.4% (95%CI 95.4–98.7) NPV, yielding 11 false negative results (*P*-values for comparison of sensitivity and NPV between the two single-draw strategies = 0.11). A single hs-cTnI determination < 5 ng/L in combination with a low-risk ECG achieved 97.8% (95%CI 95.0–99.3) sensitivity and 98.7% (95%CI 96.9–99.6) NPV, yielding five false negative results. The ESC 0/1-h algorithm ruled out MI with 98.2% (95%CI 95.6–99.5) sensitivity and 99.0% (95%CI 97.5–99.7) NPV (*P*-values for comparison of sensitivity and NPV versus the dual-marker strategy = 0.092 and 0.064, respectively; *P*-values for comparison of sensitivity and NPV versus single hs-cTnI ≥ 0.99 and 0.610, respectively), yielding four false negative results (Figure 2).

**TABLE 2:** Head-to-head comparison of safety and effectiveness.

R/O strategy	Sensitivity,%	p*	NPV,%	p**	Total R/O (FN/TN) effectiveness,%	p*
**Overall, *n* = 1,136**						
ESC 0/1-h	98.2 (95.6, 99.5)	Ref.	99.0 (97.5, 99.7)	Ref.	402 (4/398), 35.4%	Ref.
Dual 0 h	95.2 (91.5, 97.6)	0.092	97.4 (95.4, 98.7)	0.064	425 (11/414), 37.4%	0.38
hs-cTnI 0 h	97.8 (95.0, 99.3)	>0.99	98.7 (96.9, 99.6)	0.610	374 (5/374), 32.9%	0.008
**Symptom onset < 6 h, *n* = 448**						
ESC 0/1-h	96.9 (91.1, 99.4)	Ref.	98.2 (94.8, 99.6)	Ref.	165 (3/162), 36.8%	Ref.
Dual 0 h	92.7 (85.6, 97.0)	0.34	95.8 (91.6, 98.3)	0.200	167 (7/160), 37.3%	0.93
hs-cTnI 0 h	96.9 (91.1, 99.4)	>0.99	98.0 (94.3, 99.6)	0.920	152 (3/149), 33.9%	0.072
**Symptom onset < 3 h, *n* = 321**						
ESC 0/1-h	95.8 (88.1, 99.1)	Ref.	97.6 (93.0, 99.5)	Ref.	123 (3/120), 38.3%	Ref.
Dual 0 h	94.4 (86.2, 98.4)	>0.99	96.6 (91.6, 99.1)	0.670	119 (4/115), 37.1%	0.66
hs-cTnI 0 h	95.8 (88.1, 99.1)	>0.99	97.4 (92.5, 99.5)	0.920	114 (3/111) 35.5%	0.14
**Symptom onset < 1 h, *n* = 87**						
ESC 0/1-h	100.0 (83.2, 100.0)	Ref.	100.0 (89.1, 100.0)	Ref.	32 (0/32), 36.8%	Ref.
Dual 0 h	95.0 (75.1, 99.9)	>0.99	96.9 (83.8, 99.9)	0.310	32 (1/31), 36.8%	>0.99
hs-cTnI 0 h	95.0 (75.1, 99.9)	>0.99	97.0 (84.2, 99.9)	0.320	33 (1/32), 37.9%	>0.99
**GRACE < 140, *n* = 941**						
ESC 0/1-h	97.6 (93.9, 99.3)	Ref.	99.0 (97.4, 99.7)	Ref.	388 (4/384), 41.2%	Ref.
Dual 0 h	93.9 (89.1, 97.0)	0.15	97.5 (95.5, 98.8)	0.096	406 (10/396), 43.1%	0.51
hs-cTnI 0 h	97.0 (93.0, 99.0)	>0.99	98.6 (96.8, 99.6)	0.630	365 (5/360), 38.8%	0.025
**eGFR < 60, *n* = 288**						
ESC 0/1-h	98.8 (93.2, 100.0)	Ref.	97.3 (85.8, 99.9)	Ref.	37 (1/36), 12.8%	Ref.
Dual 0 h	95.0 (87.7, 98.6)	0.37	88.6 (73.3, 96.8)	0.140	35 (4/31), 12.2%	0.55
hs-cTnI 0 h	100.0 (95.5, 100.0)	>0.99	100.0 (86.3, 100.0)	0.400	25 (0/25), 8.7%	0.035

* McNemar test as proposed by Lipinski et al. (17); ** Raskovalova et al. (16); R/O, rule-out.

Overall, the two instant, single-sampling strategies were comparably safe as the ESC 0/1-h dual-sampling algorithm, and both sensitivity and NPV were consistent across various subgroups, including early-comers (with symptom onset < 6 h, < 3 h and < 1 h), GRACE score < 140 and eGFR < 60 ml/min/1.73 m^2^ (Table 2).

### Effectiveness

Overall, the percentage of patients triaged to the rule-out zone were similar for the dual-marker strategy and the ESC 0/1-h algorithm in the overall cohort and across subgroups. The single hs-cTnI concentration strategy ruled out a significantly lower percentage of patients (32.9%) compared with the two other strategies (dual-marker 37.4%, *P* < 0.001; ESC 0/1-h 35.4%, *P* = 0.013), both in the overall cohort and in the GRACE score < 140 and eGFR < 60 ml/min/1.73 m^2^ subgroups (Table 2 and Figure 2).

### Prognostic performance

Over a median follow-up period of 12 months, the cumulative incidence rate of the primary composite endpoint of all-cause death and MI was similar across different strategies (1% for the dual-marker strategy, 0.84% for the single hs-cTnI approach and 1.1% for the ESC 0/1-h algorithm; *P* = NS for all pairwise grouped Jackknife tests) (Figure 2).

**FIGURE 3:**
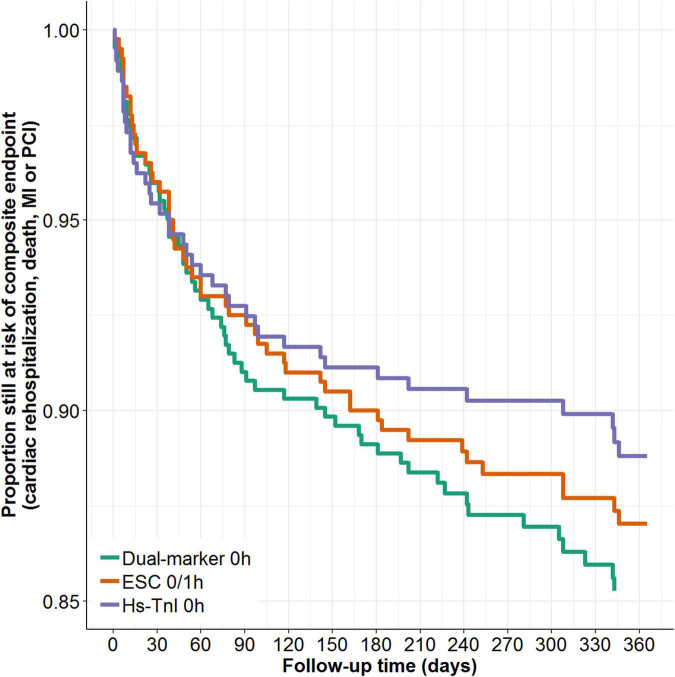
Kaplan-Meier analysis of the 12-month secondary composite endpoint by different rule-out strategies.

Conversely, the cumulative incidence rate of the secondary composite endpoint of all-cause death, MI, subsequent PCI and cardiac hospitalization was lowest for the single hs-cTnI concentration algorithm (11.2% for the single hs-cTnI approach; 14.4% for the dual-marker strategy; 12.1% for the ESC 0/1-h algorithm and; pairwise grouped jackknife tests: *P* = 0.023 for ESC 0/1-h vs. hs-cTnI 0 h; *P* = 0.72 for dual-marker 0 h vs. ESC 0/1-h; *P* = 0.008 for dual-marker vs. single hs-cTnI) (Figure 3).

## Discussion

The two single-sampling instant rule-out pathways tested in this study, one based on a dual-marker strategy combining 2nd generation us-Cop and hs-cTnI, and the other based on one-off hs-cTnI determination, can both be considered as safe alternatives to the ESC hs-cTn 0/1-h strategy for the triage of suspected AMI in patients with a low-risk ECG. Overall, the two instant algorithms showed similar safety for the rapid rule-out of AMI, also yielding similar prognostic information. While the single-sampling hs-cTnI strategy proved to be slightly, but significantly, less effective than the single-sampling dual-marker strategy, it was associated with a numerically lower rate of false negative results and with fewer downstream cardiac hospitalizations and revascularization procedures.

Our findings corroborate previous data on the diagnostic and prognostic yield of competing rule-out strategies for MI (10, 32–36), providing evidence on their comparable clinical performance regardless of time of symptom onset, GRACE risk score and renal function status.

In general, in the setting of increasingly overcrowded ED with long waiting times and potentially high 7-day mortality (37), safe and effective pathways for the instant rule-out of MI appear relevant clinical achievements. The current standard is the ESC guidelines-endorsed rapid 0/1-h algorithm using low cut-off hs-cTn concentrations, the safety and effectiveness of which was tested in different cohorts (21, 38). A drawback is here, however, the need for a second venipuncture and the 1-h waiting for retesting. Single-sampling approaches would allow an immediate decision, accelerate medical triage, potentially save costs and staff time, and reduce the ED length of stay (39). One such approach is the combination of cTn and copeptin. Regarding this, the BIC-8 RCT showed that it efficiently identifies patients who can be safely discharged to outpatient care, with the potential of shortening patients’ permanence in the ED at no added risk for adverse outcomes (10). Findings from the BIC-8 trial have been also recently confirmed by independent and real-world data from the prospective, multicenter, European Pro-Core registry (15). The ongoing Accelerated Rule Out of Myocardial Infarction (AROMI) trial (clinicaltrials.gov: NCT02666326) is currently investigating, in an open randomized setting, the safety of such prehospital MI rule-out protocol. Recently, several publications have also suggested the use of a single-sampling instant rule-out approach using hs-cTn concentrations below the 99th percentile (6, 7, 34). The High-STEACS investigators previously validated a single admission hs-cTnI concentration < 5 ng/L using the Abbott Architect hs-cTnI (40). A recent individual patient-level meta-analysis in 22,457 patients further demonstrated that a hs-cTnI concentration < 5 ng/L at presentation yields a NPV as high as 99.5% for MI or cardiac death at 30 days (6). Both these instant rule-out approaches will be especially beneficial when the availability of point-of-care assays will open the opportunity for prehospital triage of chest pain (41).

### Comparison of the different approaches here tested and practical considerations

Both single-sampling approaches performed similarly to the ESC 0/1-h hs-cTn protocol: one uses the combination of hs-cTn with us-Cop, the other a particularly low hs-cTn threshold. However, beyond the apparent clinical equipoise, the dual marker strategy would appear more complex than the hs-cTnI approach because requiring the assay of two analytes with ensuing practical and cost issues related to having the additional us-Cop test available. Furthermore, the dual marker strategy did result in a higher rate of false negative results in our cohort, yielding numerically lower sensitivity and NPV compared with both single-marker strategies, with a trend toward a poorer diagnostic performance. However, if a sensitivity > 99% and an NPV > 99.5% are needed, none of the tested algorithms would have the required diagnostic performance (42). But is this truly required in practice? Arguably not, and indeed very few tests in clinical practice have achieved such a goal (43).

A few considerations, however, lead to a more nuanced appraisal. One consideration pertains in general to all single-sampling approaches compared to the dual-sampling approach: wider system factors, such as prolonged waiting times and high volume loads of the ED, variably affect the clinical effectiveness of alleged “instant” rule-out strategies (23). As an example, the multi-center LoDED RCT – although demonstrating the effectiveness of an instant rule-out strategy based on a single undetectable hs-cTn test taken on arrival at the ED together with a normal ECG – failed to meet the primary endpoint (discharge from the hospital within 4 h from arrival), as discharge time was not statistically different from the usual care dual-sampling pathways that already incorporate hs-cTn tests (23).

As to the direct comparison of the two single-sampling strategies here assessed:

1. The overall implementation of hs-cTn assays in general and the use of accelerated diagnostic protocols is lagging behind globally (44). While the dual-marker strategy was here in this study investigated using a hs-cTnI assay, a higher added benefit has been reported when the more widely available conventional or contemporary sensitive assays are used (17).
2. Both the ESC 0/1-h protocol and the single-measurement strategy using a hs-cTnI with < 10% CV at the decision cutoff level used assay-specific thresholds that have to be carefully confirmed after individual validation for each of the several commercially available hs-cTn assays available.
3. Changes in practice that result from the use of rapid discharge protocols may all potentially be associated with late excess death or MI rates, as recently disclosed from a *post hoc* analysis of the RAPID-TnT trial (45). Although the use of a 0/1-h algorithm has proven capable of expediting the ED discharge of patients with low event rates at 30 days, an increase in death or MI at 1 year was observed in those with unmasked hs-cTnT concentrations. Particularly, among patients with intermediate cardiac troponin concentrations, where management was informed by a 0/1-h unmasked hs-cTnT, more frequent revascularization procedures and fewer non-invasive cardiac investigation were observed, questioning the downstream management of intermediate-risk patients. In the HiSTORIC trial (20, 22) – a stepped-wedge cluster RCT that evaluated the implementation of a hs-cTnI assay in 31,493 patients presenting with suspected acute coronary syndrome across 10 secondary and tertiary hospitals in Scotland – all-cause mortality was > 5% and the reattendance rate was about 39% at 1 year, independent of the standard-care or early rule-out pathways used. We should therefore not overlook the importance of life-threatening conditions other than cardiovascular disease at the triage of chest pain, and in this regard it is important to consider that copeptin confers prognostic information that is complementary to cTn in general, both in various acute cardiovascular settings – including acute coronary syndrome, heart failure, and acute pulmonary embolism – where cardiac injury occurs, as well as in a variety of potentially life-threatening non-cardiac conditions, including acute gastrointestinal diseases, bleeding, infections or neurological disorders (15, 46). Recent evidence further supports the concept that prognostic implications of copeptin are not only mediated by heart failure or endogenous stress, but are in the trajectory of increased general vulnerability of the organism (47).
4. The selection of immediate rule-out cut-offs lower than the assay-specific upper reference limit (i.e., functional sensitivity) may be extremely variable in terms of reproducibility, and even a very slight error in determination might here imply different NPV and effectiveness. This would appear to be more relevant for the single-marker approach based only on hs-cTn than for the dual-marker approach, where the rule-out would depend on the results of two rather than on one single marker.

### Limitations

We recognize limitations of our study. Firstly, our findings are limited to specific copeptin and troponin assays and cannot be generalized to other assays. Secondly, our results derive from a prospective single-center study, and therefore require external validation. Thirdly, no patients were managed on the basis of the assays performed, and differences in clinical management and follow-up might have influenced outcomes and might be different in a prospective evaluation of discharge strategies. However, previous evidence from RCT and prospective real-world data have already documented the safety and effectiveness of the dual-marker strategy (10, 15). Fourthly, despite current literature strongly identifies the existence of sex-driven differences in hs-cTn levels in reference populations, we did not endorse sex-specific cut-offs in our analyses, essentially because their adoption is still debated, because of the paucity of data on the underlying pathophysiology, and because of the current uncertainty on the advantages than this could have on the management and prognosis of acute coronary syndromes in women. Finally, further research is warranted to assess and compare the cost-effectiveness of different rapid rule-out strategies, balancing the extra cost and organizational complexity of a dual-marker strategy, with the time-saving and efficiency of the patient discharge system.

## Conclusion

In patients with a low-risk ECG, instant rule-out pathways with either a dual-marker test using high-sensitive troponin and copeptin, or a single high-sensitivity cardiac troponin test offer reasonable diagnostic strategies alternative to the ESC hs-cTnI 0/1-h-algorithm, allowing safe triage of patients presenting to the ED with suspected AMI. However, adding copeptin to hs-cTn does not improve the safety of instant rule-out when compared to single rule-out hs-cTn values.

## Data availability statement

The raw data supporting the conclusions of this article will be made available by the authors, without undue reservation.

## Ethics statement

The studies involving human participants were reviewed and approved by Universitätsklinikum Hamburg-Eppendorf. The patients/participants provided their written informed consent to participate in this study.

## Author contributions

FR, JN, NS, TZ, SB, DW, and RD: study concept and design. FR, JN, NS, IC, RD, MZ, and DW: drafting of the manuscript. FR, JN, NS, FO, NR, and DW: statistical analysis. DW: obtained funding. JN, TZ, and DW: administrative, technical, and material support. SB, RD, and DW: study supervision. FR, JN, NR, NS, FO, IC, TZ, SS, TH, MG, SP, MZ, SB, DW, and RD: acquisition, analysis, interpretation of data, and critical revision of the manuscript for important intellectual content. All authors contributed to the article and approved the submitted version.

## Footnotes

1
www.R-project.org
